# Implementing an intrapartum package of interventions to improve quality of care to reduce the burden of preterm birth in Kenya and Uganda

**DOI:** 10.1186/s43058-021-00109-w

**Published:** 2021-01-28

**Authors:** Gertrude Namazzi, Kevin Abidha Achola, Alisa Jenny, Nicole Santos, Elizabeth Butrick, Phelgona Otieno, Peter Waiswa, Dilys Walker, Preterm Birth Initiative Kenya & Uganda Implementation Research Collaborative, Alejandra Benitez, Ryan Keating, Felicia Lester, Rikita Merai, Lara Miller, Roger Myrick, Nancy Sloan, Darious Kajjo, Ayub Kakaire, Paul Mubiri, Lawrence Kazibwe, Harriet Nambuya, Phillip Wandulu, Angela Namala, Leah Kirumbi, Nelly Mugo, Grace Nalwa, Beatrice Olack, Christopher Otare, Anthony Wanyoro

**Affiliations:** 1 School of Public Health, College of Health Sciences, Makerere University, Kampala, Uganda; 2 Kenya Medical Research Institute, Nairobi, Kenya; 3 Institute for Global Health Sciences, University of California, San Francisco, USA; 4 Department of Obstetrics, Gynaecology and Reproductive Sciences, University of California, San Francisco, USA

**Keywords:** Implementation process, TIDieR framework, Preterm birth, Quality of care

## Abstract

**Background:**

Quality of care during the intrapartum and immediate postnatal period for maternal and newborn health remains a major challenge due to the multiple health system bottlenecks in low-income countries. Reports of complex interventions that have been effective in reducing maternal and newborn mortality in these settings are usually limited in description, which inhibits learning and replication. We present a detailed account of the Preterm Birth Initiative (PTBi) implementation process, experiences and lessons learnt to inform scale-up and replication.

**Methods:**

Using the TiDieR framework, we detail how the PTBi implemented an integrated package of interventions through a pair-matched cluster randomized control trial in 20 health facilities in Migori County, Kenya, and the Busoga region in east central Uganda from 2016 to 2019. The package aimed to improve quality of care during the intrapartum and immediate postnatal period with a focus on preterm birth. The package included data strengthening (DS) and introduction of a modified WHO Safe Childbirth Checklist (mSCC), simulation-based training and mentoring (PRONTO), and a Quality Improvement (QI) Collaborative.

**Results:**

In 2016, DS and mSCC were introduced to improve existing data processes and increase the quality of data for measures needed to evaluate study impact. PRONTO and QI interventions were then rolled out sequentially. While package components were implemented with fidelity, some implementation processes required contextual adaptation to allow alignment with national priorities and guidelines, and flexibility to optimize uptake.

**Conclusion:**

Lessons learned included the importance of synergy between interventions, the need for local leadership engagement, and the value of strengthening local systems and resources. Adaptations of individual elements of the package to suit the local context were important for effective implementation, and the TIDieR framework provides the guidance needed in detailed description to replicate such a complex intervention in other settings. Detailed documentation of the implementation process of a complex intervention with mutually synergistic components can help contextualize trial results and potential for scale-up. The trial is registered at ClinicalTrials.govNCT03112018, registered December 2016, posted April 2017.

**Supplementary Information:**

The online version contains supplementary material available at 10.1186/s43058-021-00109-w.

Contribution to literature

- Randomized intervention trials often have limited descriptions of the detailed implementation process to enable replication. Results from the PTBi CRCT showed a significant reduction of fresh stillbirth and mortality among eligible infants in a low-resource setting, and the details for replicability are a critical and complementary part of the study findings.
- This paper provides a detailed account of a complex intervention, which is important particularly to inform decision-makers in low-resource settings about reproducibility and scale-up.
- These findings contribute to the growing acknowledgement of the importance of using a standardized framework such as TIDieR to ascertain key criteria needed to adopt these types of evidence-based packages for maternal and neonatal health.

## Background

Over 80% of the estimated 15 million preterm babies born each year occur in sub-Saharan Africa and South Asia [1, 2]. Complications due to prematurity are the leading cause of neonatal and under-five mortality in low- and middle-income countries [3]. Quality of care (QoC) during birth and the immediate postnatal period is believed to be the most important window of opportunity for interventions to reduce preventable maternal and newborn deaths in these settings [4]. An estimated three quarters of preterm neonatal deaths could be averted by implementing low-cost evidence-based interventions at the time of birth, including essential newborn care, provision of antenatal corticosteroids to eligible mothers with preterm labor, Kangaroo Mother Care (KMC), support for breastfeeding, and appropriate administration of antibiotics for newborn infections [5, 6]. Another one million preterm stillbirths could be averted each year by improved intrapartum care [7]. Addressing the QoC gap is critical for achieving the ambitious targets set by the Sustainable Development Goals [4].

Some authors have proposed approaches to QoC improvement around the time of birth that are integrated and address the complexity of the particular care ecosytem, including provider skills and knowledge, system readiness, and experience of care [8–11]. However, reports of successful QoC intrapartum interventions are limited and often describe single discrete approaches [12–14]. QoC improvement efforts that are multi-dimensional and adapted to fit a particular context are rare and often insufficiently described to enable learning either for replication or to inform scale-up.

The Template for Intervention Description and Replication (TIDieR) framework provides an approach to document detailed implementation of both intervention and control arms of a study trial [15]. The framework was developed by an international group of experts in order to support comprehensive reporting of interventions for better understanding of implementation processes and their replicability. The 12-item TIDieR checklist includes name; rationale or theory of the study elements; materials used; who, how, where, when, and how much of the intervention element was provided during the implementation; and any tailoring and modifications, including the extent of adherence to the planned intervention. Use of the TIDieR checklist is a systematic way of reporting implementation of complex interventions and has gained usage recently [16].

The East Africa Preterm Birth Initiative (PTBi-EA) addressed the complexity of QoC by implementing a multi-component intrapartum and immediate newborn care quality improvement package in rural facilities in Kenya and Uganda, between 2016 and 2019. The primary results of this cluster randomized control trial (CRCT) found a 0.66 odds ratio (CI, 0.54–0.81) for the combined primary outcome indicator of fresh stillbirths and 28-day mortality among eligible infants [17]. This paper aims to describe the implementation of the intervention package in detail using the TIDieR framework [15]. We report the implementation process, adaptations, experiences, and lessons learnt in order to inform local scale-up, or replication in other contexts.

## Methods

Per the TIDieR checklist, the intervention name, rationale, and locations (items #1, 2, and 7) are described in the “Methods” section, while the intervention activities, mode and frequency of delivery, adaptations, modifications, and fidelity (i.e., items 3–6 and 8–12) are presented in the “Results” section. A populated TIDieR checklist is provided as Additional file 1.

### Study sites and study design

We conducted a pair-matched CRCT in 5 districts in the Busoga Region of east central Uganda (Jinja, Kamuli, Iganga, Bugiri, and Mayuge) and Migori County, in south west Kenya, among 20 public sector and private-not-for profit health facilities. The Busoga region has a population of about 3 million people, 80% of whom live in rural areas and practice subsistence farming. The Busoga regional neonatal mortality and stillbirth rates are similar to the national rates of 27/1000 live births and 21/1000 total births, respectively [18]. Migori County has a population of about 1 million people with neonatal mortality and stillbirth rates of 19/1000 live births, and 9.8/1000 total births, respectively [19]. The preterm birth rates are estimated at 14% in Busoga and 12% in Migori County [18, 19].

**Fig. 1:**
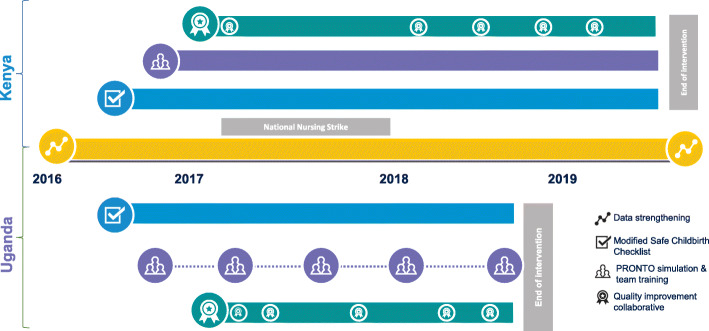
PTBi intervention implementation roll-out

Due to the study design, sample size targets, and workforce strikes in Kenya, the two countries had different timelines, though they began at the same time. The intervention period in Uganda was from October 1, 2016, to May 31, 2018, while in Kenya it was from October 1, 2016, to April 30, 2019 (Fig. 1). Among the 20 facilities included in the study, 4 were in Uganda and 16 were in Kenya, totaling 28,174 and 25,746 enrolled births during the study period, respectively. In Uganda, 2 facilities were public and 2 were missionary general hospitals. In Kenya, 14 were public and 2 were missionary hospitals. All study facilities offer maternity services 24 h a day, and 7 had cesarean capacity. In both countries, maternity wards are staffed predominantly by nurses and nurse-midwives. Higher level facilities generally have 2–3 midwives per shift, whereas lower level facilities may have only 1, often with significant additional duties. Selection and matching criteria of the 20 study sites were based on delivery volume, type of hospital, number of providers, and newborn outcome rates. These details are described in the study protocol published elsewhere [20].

Ten facilities from matched pairs (8 from Kenya and 2 from Uganda) were randomly selected to receive the complete intervention package, as were 3 larger referral hospitals—the county referral hospital in Kenya, the regional referral hospital in Uganda, and one district hospital in Uganda that serves as a de-facto referral hospital for other district hospitals in the study. These hospitals were determined to not have a comparable pair and were excluded from the randomization process and primary analysis.

### The intervention package

The intervention package included maternity register data strengthening (DS), introduction of a modified WHO Safe Childbirth Checklist (mSCC), capacity building in emergency obstetric and neonatal care using PRONTO simulation training and mentorship, and creation of a Quality Improvement (QI) Collaborative aiming to improve intrapartum and newborn care, particularly among preterm births. All 20 study facilities received DS and mSCC components to foundationally improve data quality, standardize definitions of key indicators related to gestational age (GA) and newborn outcomes, and reinforce critical EBPs. Intervention sites received additional mentorship and support of these two interventions through synergies with PRONTO simulation and team training and QI Collaborative components. A summary graphic describing the package by arm can be found elsewhere [17].

Before any interventions were introduced, we conducted health facility assessments in December 2015 and January 2016 for Kenya and Uganda, respectively. These assessments helped us select which facilities could accommodate this intervention package and what needed to be “added” in order for the site to be included in the study. The initial facility assessments uncovered critical gaps in equipment and supplies. These investments, while critical, were relatively small in scale and did not exceed US$50,000 in either country and were provided to both intervention and control facilities. Since these items were only provided at the start of the study, they were not considered part of the intervention package. A description of all equipment and supplies is provided in Additional file 2.

### Rationale of the intervention package

In Uganda, neonatal mortality has barely changed over the past 15 years. The neonatal mortality rate is 27/1000 live births despite increased facility delivery from 38 to 77% during the same period [18]. This points to challenges in QoC provision during labor and the immediate postnatal period. Previous studies carried out in Uganda revealed limitations in availability of essential commodities, lack of skills for maternal, and newborn health care of the frontline health providers, staffing limitations particularly in the neonatal care unit, and structural challenges [21–23]. In Kenya, although neonatal mortality has steadily declined, from 31 deaths per 1000 live births in 2004, to 22 deaths per 1000 in the 5 years that preceded survey year 2014, the decline is not similar across regions [19]. This may be related to differences in improvement of maternal and newborn health care services.

The intervention package was developed based on available evidence that up to two thirds of neonatal deaths could be saved by improved uptake of EBPs described by the Every Newborn Action Plan (ENAP) [24]. Previous scholars recommend use of priority packages in an integrated approach for effective improvement in neonatal outcomes [25, 26]. Each of the PTBi package components was chosen because it met stakeholder requirements of (1) having the potential to improve QoC for preterm infants and for the mother-baby dyad more generally and (2) existing evidence demonstrating effectiveness as an individual intervention.

Our theory of change is documented in the published protocol [20]. The PTBi package was designed to provide a strong foundation of data for clinical decision-making and adaptive learning to reinforce EBPs, strengthened by improved provider skills and teamwork, and continuous facility-based QI. Data quality is essential for any trial; thus, our package emphasized collection and utilization of quality data from already existing data systems. By introducing these interventions together, we hypothesized that they would mutually reinforce each other to create an enabling environment for change.

## Results

**Fig. 2:**
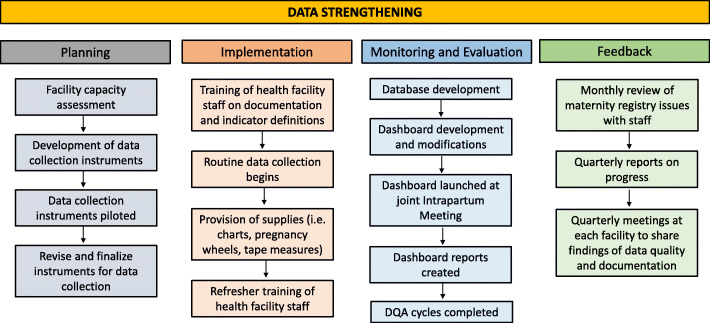
Data strengthening implementation process: a Program Impact Pathway

During the study period, each country used a Program Impact Pathway (PIP) [27] for each of the intervention components to identify and map all of the elements needed to successfully implement them. While each country adapted the PIP to meet its specific needs, each consisted of four phases: a planning phase, an implementation phase, a monitoring and evaluation phase, and a feedback phase. Figure 2 provides an example PIP.

### Data strengthening (DS)

#### Planning

Rather than introducing parallel data systems, we committed to using an existing data system, namely maternity registers. Improvements in consistency, completeness, and maternity register data use in the study sites were critical to establishing baseline measures and to achieving a demonstrable impact on preterm survival. In addition, we felt that improving these measures in existing data sources would bring sustainable change to our sites, as outlined in a whiteboard video we created to teach providers about the importance of accurate maternity register data [28].

We conducted an initial Data Quality Assessment (DQA) during the pre-trial facility assessments. We documented what components of the existing data systems warranted additional investment and targeted strengthening [29]. These findings were used to inform the DS training curriculum.

#### Implementation

The DS team included 5 data specialists in Kenya and 2 in Uganda. The initial DS training lasted for 2 days in Kenya and 3 days in Uganda in May 2016. DS training included an overview of the health information system and guidelines with facility clinical leadership, health records officers and hospital maternity in-charges, followed by site trainings with maternity nurses and health records staff. We also introduced standard tools to improve GA assessment (GA wheels and tape measures) and reviewed indicator definitions and standardization based on national guidelines.

According to the study protocol, we planned a single DS training at the start of the study; however, after the initial DS training, most providers still lacked adequate knowledge and skills in capturing the relevant data elements. Thus, we modified our efforts in all facilities. Two additional 2-day refresher DS trainings were offered during the course of the study, once per year of implementation. In Uganda, additional feedback on data quality issues, data source completion and adherence to indicator definitions was provided during the monthly data collection sessions for all facilities. In Kenya, refreshers were conducted less formally during monthly data mentoring and collection visits with staff available.

In addition, there was a lack of inpatient maternity patient charts for recording clinical notes, which are critical for patient management and monitoring QI processes. The project therefore provided a total of 79,200 and 13,000 maternity charts in Uganda and Kenya respectively, and 11,880 neonatal charts, divided into two batches for Kenya and three for Uganda, based on annual utilization of each facility to ensure availability of patient records. In Uganda, the maternity chart was developed based on the existing Ministry of Health (MoH) charts used at the national referral hospital, while we adopted the neonatal chart previously used by the Uganda Pediatric Association for neonatal surveillance in newborn care units. In Kenya, the county’s standard chart was provided.

#### Monitoring and evaluation/feedback

We worked with in-country stakeholders to develop and iterate a Data Dashboard to improve data use and dissemination of our study data. During the intervention phase, the DS team visited all facilities monthly to share dashboard reports and review data with data officers, providers, and maternity ward in-charges.

DQAs were conducted after every 6 months, twice in Uganda, and three times in Kenya to collect data on specific DHIS-related indicators and assess gaps between reported and actual indicators (e.g., errors in transcription) across all control and intervention facilities. We followed the DQA approach utilized in HIV treatment data monitoring systems [30].

### Modified safe childbirth checklist (mSCC)

#### Planning

We used the WHO Implementation Guide for the Safe Childbirth Checklist to guide our approach to adaptation and roll-out of the checklist [31]. The PTBi study team convened teams of critical stakeholders in Kenya and in Uganda to modify the mSCC with a focus on preterm labor and birth. The goal was that it serves as both a decision aid for providers to appropriately use EBPs during triage, admission, intrapartum, and immediate postnatal period, as well as a data source for the study. We convened half-day meetings to align the mSCC with national guidelines and to include items for a greater focus on identification of preterm labor, documentation of GA, and management of preterm birth. An additional pause point was added in each country to better assess women (particularly GA) who present at maternity and are checked (triaged) but may or may not be admitted. In addition, specific indications for appropriate use of antenatal corticosteroids (ACS), immediate skin to skin, and KMC were added. Each country team adapted the checklist to their local context (Table 1).

**Table 1:** A summary of PTBi intrapartum package intervention, contextual modifications, and key insights

Intervention component	Description	Who implemented	Uganda modifications	Kenya modifications	Key insights and challenges
Data strengthening	GA estimation and indicator definition review. Data quality/completeness monitoring and feedback	5 data specialists in Kenya, and 2 in Uganda	Continuous support rather than a single training	Continuous support rather than a single training	• Staff shortages, inconsistent supply of appropriate resources/reporting tools, and data storage infrastructure affected implementation • Challenge to identify and select key people to participate in data trainings, which affected reach • Facility staff’s lack of internet access to view Data Dashboard limited its utility at study sites • Bottom up approach could be better complemented by MoH engagement for sustainability prospects
Modified WHO Safe Childbirth Checklist	A modified checklist with 5 pause points and a focus on identification and care of preterm/low birthweight babies	2 clinical staff team and 2 data specialists in Uganda, 4 clinical staff team in Kenya	Split up per pause points and integrated in the maternity patient chart to make it user friendly and for consistency	Integrated into the maternity inpatient chart by being attached either at the back or in front of the patient chart. A small monetary incentive implemented	• Modification of mSCC raised profile of preterm babies • Duplicative nature between patient chart and mSCC created unnecessary documentation and burden for personnel • Financial incentive in Kenya threatened sustainability of tool • Continued use of mSCC is reliant on administration and policymakers
PRONTO simulation training and mentorship	Simulation-based training and mentorship focusing on intrapartum and immediate newborn care. Complemented by clinical bedside mentoring	10 mentors (2 nurses and 8 clinicians) in Uganda and, 5 nurses in Kenya	• Curriculum adjusted to reflect Ugandan MOH protocols and guidelines • Curriculum revised to focus on newborn care, particularly preterm care • The duration of mentorship was increased from one to two days to enable bedside mentorship • Additional clinical mentorship visits included for 2-day bedside mentorship	• Facility mentorship days reduced from 5 to 4 days per week to give room for adequate debrief and preparation for the next facility/ week work. • Curriculum aligned with the existing Kenya MOH protocols • Additional adjustments made to address (a) Birth preparedness for preterms; (b) referral management; and (c) immediate management and transfer of a preterm in a warm environment (skin-skin, KMC, warming blankets, referral systems)	• PRONTO approach and onsite mentorship enhanced the learning experience • High staff turnover/rotation affected consistency of PRONTO participation and dosages in training across curriculum • Contextual suitability and sustainability of PRONTO warrants further attention, such as training cascades (e.g., expert and resident mentors)
Quality improvement Collaborative	Model for Improvement approach: QI teams conduct PDSA cycles and share experiences in learning sessions. Indicators to track progress included: GA estimation, ACS to eligible mothers, and KMC care	1 QI coach and 10 PRONTO mentors in Uganda, 2 QI coaches in Kenya	Included process indicators of mSCC, partograph, and monitoring of sick and small newborn babies	Included process indicators mSCC, partograph monitoring, and referrals	• QI was closely linked to the 3 other interventions leading to perceived improvements in teamwork, communication and self-efficacy • Adherence to QI meeting frequency and attendance varied, underscoring importance of facilitation and management • Alignment with county and other ongoing implementation/QI efforts was critical to ensure complementarity in activities and resources

#### Implementation

Before launching the intervention package in all facilities, a pilot trial of each country’s mSCC provided additional input to optimize content and roll-out. The finalized mSCC was introduced by the clinical study teams during initial DS training activities at all 23 sites. The mSCC was included in the maternity inpatient chart or record for each woman in all facilities.

Following low response to an initial roll-out assessed at 8 months at all facilities, modifications on use of the mSCC was deemed necessary. The Kenya team decided to provide a small monetary incentive (US$.50) at each study facility (intervention and control), shared among maternity and newborn unit health workers, for each mSCC completed. In Uganda, care providers requested that the checklist be split up by pause point and integrated into the maternity patient chart, rather than be placed at the beginning or end of the chart. For example, the triage section was placed at the start, while the before discharge section was placed at the end. In Kenya, the checklist was incorporated as part of the patient chart and was placed at the front page. Subsequently, in the intervention facilities in both countries, mSCC completion was included among the QI indicators. This led to increased awareness of the importance of the mSCC and improvements in its utilization among intervention hospitals.

#### Monitoring and evaluation/feedback

Study personnel in both countries monitored mSCC completeness and uptake by each of the 5 pause points either by convenience or purposive sampling. In Kenya, study data staff reviewed all maternity charts of preterm cases eligible for follow-up each month and abstracted data from the checklists of preterm cases, where available. These data were regularly reported back to facilities, and were included in the Data Dashboard.

### PRONTO simulation-based training and mentorship

#### Planning

In both Kenya and Uganda, key stakeholders including midwives, nurses, doctors, obstetricians, MoH officials and study clinicians met to adapt the PRONTO curriculum to meet the local training gaps and address critical EBPs related to preterm labor and birth (Table 1). PRONTO is an international NGO that conducts simulation-based training, primarily focused on emergency obstetric and newborn care (EmONC) in low-resource settings, with an emphasis on team training and respectful maternity care. The core PRONTO curriculum includes management of hemorrhage, preeclampsia, birth asphyxia, and respectful maternity care. The adapted training emphasized identification and management of preeclampsia, chorioamnionitis, neonatal resuscitation, and other conditions related to preterm birth. As core PRONTO competencies, the training knowledge reviews included communication skills and teamwork activities necessary in emergency situations, in both countries as well as addressing respectful maternity care. The curriculum also included management of sepsis and hemorrhage and the main causes of neonatal and maternal mortality. The final curriculum was formatted into separate manuals for each country, and PRONTO supply kits provided to each mentor team.

Five mentors/trainers in Kenya and 10 in Uganda underwent a two-step process for becoming PRONTO mentors. The process involved an initial 5-day introduction to simulation facilitation and part one of the PTBi curriculum training, followed by a second 2-day advanced facilitator training 4 months later. In alignment with facility levels and staffing, 5 mentors (nurse and midwives) in Kenya were chosen from among 10 candidates who completed the initial training of mentors. Mentors were selected based on knowledge in obstetric and newborn care, prior EmONC training, and effective facilitation and leadership skills. Mentors had to be outgoing, empathetic, compassionate, and engaging, as well as willing to work in different facilities in the specified geographical areas. In Uganda, 20 candidates were invited to participate in training based on recommendations from stakeholder institutions and represented a diverse group of providers (midwives, doctors, and specialists) including both regional and national levels. The 10 Ugandan mentors were chosen based on knowledge in obstetric and newborn care, prior EmONC training, facilitation skills, and availability. The mentors were an interdisciplinary team of 2 nurses, 1 medical officer, 4 obstetricians, 2 pediatricians, and 1 neonatologist. Half of the mentors were from national level health facilities, while the other half of mentors were from the implementation sites to enable continuous mentorship and sustainability.

#### Implementation

The training mode of delivery varied, by design, between Kenya and Uganda. Kenya utilized an in situ mentoring program whereby each intervention facility received low-dose high-frequency/4-day per week mentorship by a pair of mentors who rotated among intervention sites during the study duration. They spent a combined total of 9–12 weeks at each intervention facility over the 31 months study duration in Kenya. Visits included bedside mentoring, video-recorded in situ simulations and debriefing, knowledge reviews, skills stations, teamwork activities, and mSCC support as well as QI roles.

In Uganda, a high-intensity/less-frequent shorter modular strategy was selected by stakeholders engaged during study planning. A modular-based training program was paired with 4 2-day long simulation refresher trainings and mentorship visits. Initially, the refresher trainings were 1-day sessions but later (during the second session) modified to 2-day long activities to allow bedside mentorship which was required for knowledge translation and skills application on patients rather than simulation alone. The 2 modules and refresher training visits were spread out during the study period, and similarly amounted to approximately 6 weeks of mentorship (Fig. 1). Thus, while the mode of delivery for training in each country was different, provider teams in each country received approximately 56–58 h of PRONTO-based instruction using the same curricular components.

Further modification in Uganda included augmentation of the PRONTO refresher simulation visits by interspersed 2-day clinical bedside mentorships which were unstructured, and based on knowledge and practice gaps identified by mentors during the visits or during previous PRONTO visits. The bedside mentorships aimed at ensuring knowledge translation and competence acquisition, in addition to re-organization of the working environment for better performance. Each clinical mentorship was run by 2 specialists—a pediatrician (or a neonatologist) and an obstetrician for Uganda.

Use of the mSCC was integrated into all PRONTO clinical activities to provide facility staff with continued opportunities to reinforce its use. Any change ideas that arose from these PRONTO activities were integrated into QI efforts.

#### Monitoring and evaluation/feedback

In addition to simulation debriefing and feedback during mentorship visits, data related to total providers trained, number of simulations, and knowledge reviews conducted were displayed on the Data Dashboards. Knowledge review data also contributed to the content for bedside clinical mentorships.

### Quality improvement (QI) collaborative

#### Planning

Each facility in the intervention arm created a designated QI team comprising facility leadership, health record staff and midwives from the labor ward, postnatal ward and the neonatal special care unit (8–12 people for Uganda and 3–5 people for Kenya). A QI technical consultant helped provide foundational training in QI methods.

#### Implementation

Individual facility QI teams carried out Plan-Do-Study-Act (PDSA) cycles. Teams met twice a month for approximately 2 h with the support of a QI coach (and a clinical PRONTO mentor for Uganda) to identify and prioritize problems or bottlenecks in the facility, determine solutions/change ideas, track changes and outcomes, and make adjustments based on the results. The QI coach was a study team member who had expertise in supporting PDSA cycles and use of QI tools, including the documentation journal, process mapping, and annotated graphs for process results tracking. The PRONTO mentor participated in QI activities and had both relevant clinical expertise and a clear understanding of the local health system.

The study allocated nominal funds (US$100 per intervention facility each month) to address stock outs of key medical supplies, items or equipment that were identified as necessary to implement the identified change ideas. However, in Uganda, due to bureaucracies in procurement it was difficult to provide the needed items in real time, thus supplies were delivered in 2 batches: in the initial facility strengthening and midway the implementation phase. In Kenya, for change ideas that needed funding, study facilities wrote short proposals that described the problem identified and the change concepts. This, upon approval by the country program leadership, was processed to procure the items needed to execute the change idea.

QI teams across facilities in each country (known collectively as the QI Collaborative) participated in a total of 5–6 joint learning sessions during the intervention period to discuss core learnings and QI indicators. A total of 5 learning sessions were conducted in each country (Fig. 1). Each learning session was followed with an activity period during which the PDSA cycles were carried out.

The first learning session marked the beginning of the QI Collaborative implementation, and involved orientation of the QI teams on the QI indicators and their definitions, sharing the baseline process data, and agreeing on the end targets. Three process indicators were identified initially as important to contribute to a decrease in neonatal mortality among preterm infants: KMC uptake, GA estimation, and use of ACS among eligible women. Modifications were made in each country by selecting additional process indicators based on national priorities and needs (Table 1). Partograph use and mSCC completeness were added in both countries. In Uganda, monitoring and feeding of preterm and low birthweight infants were added as QI indicators following one of the PRONTO mentorship visits when these practices were identified as challenges (Table 1). The Kenya QI Collaborative later added tracking of delay in neonatal and maternal emergency referral for care in one intervention and the referral facility.

During learning sessions, QI teams were taught use of the QI tools, like process mapping to identify system bottlenecks, how to develop and test change ideas, monitoring, and decision-making. QI/clinical mentors and facility leadership attended the learning sessions to identify the persistent gaps they could work on for improved performance. For example, in Uganda, more midwives were allocated to the neonatal special care unit in 3 health facilities, while in 2 other facilities, bigger rooms closer to maternity wards were allocated to be used as neonatal special care units.

Elements of the package, specifically the Data Dashboard, PRONTO, and the mSCC were integrated with QI efforts. First, the Data Dashboard generated figures to display the data visually, which better informed facility teams on progress, and highlighted remaining performance gaps. Second, areas of improvement were identified through PRONTO training and mentorship, which were shared with the QI teams. Lastly, the mSCC served as a data source to document selected QI indicators.

#### Monitoring and evaluation/feedback

QI materials included the documentation journal for tracking progress of performance of various indicators. The data sources for QI indicators included the patient charts, the maternity registers, and the mSCC. QI data were displayed on the Data Dashboard and shared monthly with data officers, care providers, and maternity ward in-charges.

### Intervention package fidelity

We achieved fidelity to the expected number of trainings for the intervention components as initially described in the protocol [20]. Specifically, DQAs were to be conducted every 6–12 months, QI learning sessions every 3–6 months, 5 PRONTO modular/refresher trainings in Uganda, and 6 weeks per mentor pair were intended in Kenya. We did not have specific measures of fidelity for the mSCC as it was designed as an optional clinical aid for providers. For DS, QI, and PRONTO, the study met or surpassed these objectives. During Uganda’s 18-month study period, 2 DQAs, 7 PRONTO trainings, and 5 QI learning sessions were convened, while 3 DQAs, 12 weeks of bedside mentorship per facility, and 5 QI learning sessions were held in Kenya over the 31-month study period (inclusive of strike). Although no fidelity benchmark was set for the mSCC, monitoring revealed that among audited records that had checklists, there were varying completion rates by pause point ranging from 75 to 90% completion.

We have summarized additional measures of fidelity in Table 2, including proposed versus delivered dose, fidelity measures captured and from what data sources, and outputs either completed or forthcoming. Additional analyses are underway to examine each individual intervention component’s process or outcome data, with manuscripts forthcoming.

**Table 2:** Dose and measurement of intervention fidelity

Intervention	Proposed dose	Delivered dose	Fidelity measures and sources	Outputs
**Data strengthening**	• Initial training • Monthly data collection and review sessions • DQAs every 6 months	• Initial training • Monthly data sessions, approx. 20 h/year • 2 DQAs in Uganda, 3 in Kenya	• Training rosters • Monitored completeness of monthly data on project dashboard • DQA reports	• Published paper documenting increased completion rates after initial data strengthening published^a^ • Paper in press on data strengthening over the life of the project in Kenya
**Modified WHO Safe Childbirth Checklist**	• Initial training • Checklists supplied for all births	• Initial training • Checklists supplied for all births	• Training rosters • Program records • Periodic audits to assess completion rates by pause point	• Completion audits showed completion rates of 75–90% by study end • mSCC-specific paper forthcoming
**PRONTO simulation training and mentorship**	• 5 trainings in Uganda • 12 weeks of in situ training/mentoring in Kenya	• 7 trainings in Uganda plus 4 beside mentoring sessions • 12 weeks of in situ training/mentoring in Kenya	• Training rosters • Pre-post knowledge tests at initial and final training sessions • Key simulations video-recoded and coded for uptake of evidence-based practices	• All facilities completed required simulations • PRONTO-specific paper on changes in knowledge and evidence-based practice in recorded simulations forthcoming
**Quality improvement Collaborative**	• Initial training • Coaching visits/ team meetings at facilities every 2 weeks • 3–6 total Collaborative learning sessions per country	• Initial training • 5 QI Collaborative sessions per country	• Program records • Coaches logged visits and topics during meetings • Collaboratives tracked indicators across facilities at each meeting	• All teams had indicators to present and change efforts to report at each collaborative. • Indicators improved in each country collaborative • QI-specific paper on QI team process and results forthcoming

^a^Keating et al. [29]

## Discussion

The PTBi CRCT yielded significant results—a 34% reduction in the odds of combined fresh stillbirth and neonatal mortality among eligible neonates in the intervention facilities, relative to the control facilities [17]. Understanding the implementation process by which this was achieved is critical. Using the TiDieR checklist as a framework, we have provided a detailed report of how we implemented this intrapartum and immediate newborn package in Kenya and Uganda. Overall, each intervention component’s content was delivered as intended; however, actual implementation processes varied from the original protocol, as expected, to contextualize and adapt to local contexts (Table 1). This level of detail provides insight for those interested in implementing a complex intervention package, as well as a framework for understanding where adaptations are helpful or even necessary. Through this exploration, we identified 3 overarching lessons learned: (1) the mutually reinforcing nature of the interventions, (2) the importance of local leadership engagement and adaptation of the interventions, and (3) the importance of considering and strengthening existing resources in the health system.

**Fig. 3:**
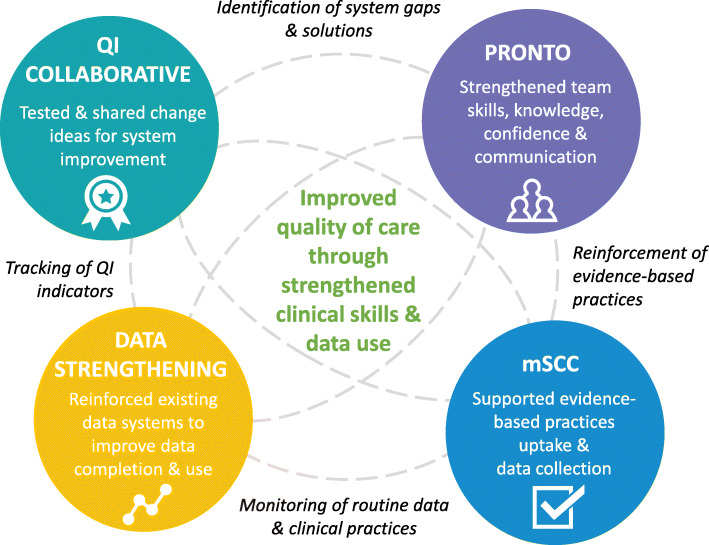
Package integration and synergy. QI, Quality Improvement; mSCC, modified Safe Childbirth Checklist

First, EBPs promoted through an intervention package with multiple mutually reinforcing components (Fig. 3) can offer an opportunity to improve the clinical care for mothers and babies in a resource-constrained setting. We found that the complex integrated package of DS, the mSCC, provider simulation-based PRONTO training, and the QI Collaborative approach was effective in continually reinforcing the importance of these EBPs from differing perspectives. For example, maternity registers served as data sources for monthly DS feedback and DQA activities. If GA data were incomplete or inaccurate, the study team would flag this as an area of improvement. GA accuracy and completion would then be reinforced by facility leadership and study mentors using the mSCC and continued DS activities. Appropriate GA assessment and related clinical decision-making would be also discussed and modeled during PRONTO trainings and bedside mentoring sessions. These activities would then relate to QI indicators (e.g., provision of ACS to mothers in preterm labor, KMC uptake). Collectively, the intentional interplay among these implementation and monitoring and evaluation/feedback activities provided a strong foundation for data use and adaptive learning to reinforce EBPs.

Second, local leadership engagement and adaptation of the intervention package were important both to align with national guidelines and priorities and to increase local ownership. In our case, leadership from both medical and political domains, and engagement from other national and regional stakeholders right from the start was critical. During the planning phase of each intervention component, key stakeholders were engaged. For example, key members of the national cadre of EMONC trainers, professional societies, the country’s first neonatologist, and regionally based obstetricians and pediatricians were all included in the PRONTO curriculum development consultation and invited to train as mentors. In addition, during implementation activities, local leadership was invited to PTBi trainings and QI Collaborative learning sessions, as well as special events about prematurity. Their involvement improved the general awareness of the burden of preterm birth among leaders, placing prematurity on top of their newborn health agenda. Local ownership by facility-based staff also helped to effectively integrate the interventions and address system bottlenecks, such as the infrastructure and staffing for the neonatal care units. QI change ideas, for example, catalyzed system improvements which required approval from facility administrators, such as relocating neonatal special care units closer to the maternity ward. During this process, local champions emerged and helped in accelerating the uptake of EBPs, at the facility and system level. These champions were critical to the success of a program and efforts should be made to identify and mentor champions at all levels of the system early on.

While we were thoughtful in the planning, implementation, and evaluation of each of the components from the start, we designed the package to be flexible in nature to enable adaptation and responsiveness to each country’s needs and health system challenges. Each component of the intervention underwent several adaptations, which were important to align the program with national strategies and the local context (Table 1). For example, co-creating the PRONTO training curriculum and piloting of the mSCC before introduction helped in acceptance and integration. Flexibility allowed us to identify areas requiring additional support, such as the need for continuous DS activities throughout the study. In some instances, health system gaps were identified by our intervention, allowing them to be addressed in real time through enhanced mentorship and QI change ideas; however, some challenges emerged that were beyond the scope of our program. For instance, stock outs of critical medical supplies and medicines are important bottlenecks to quality improvement and require system-level strategies. Lastly, there were unforeseen events that impacted the implementation process like the nurses’ strike in Kenya. The flexible nature of the intervention was important in addressing such an event, by extending the implementation period.

Our third lesson highlights the importance of working with and strengthening existing health system resources, so as to enhance local ownership and potential for replicability. We believe relying on and strengthening the existing facility registers as the primary data source is an important strength and has had lasting impact. This effort contributed to establishing a more reliable and sustainable system for monitoring outcomes, which has been documented by other QI initiatives [32]. Furthermore, as an implementation research project, we did not set up a parallel data collection system; rather than have intensive external staff collect data that is exclusively for research consumption, our efforts focused on strengthening facility data for the people who use it most. By investing in facilities and their existing staff, our approach promoted a more complete understanding of what data mean and how they can act on it. While data accuracy and completeness were a challenge even with dedicated DS efforts, PTBi’s efforts have highlighted an area for continued action and future investment.

It is worth noting that this intervention took on a regional approach through a network of facilities as collaborators. While this was part of the design for practical research reasons, it may have had unintended benefits in strengthening the relationships among facilities. This collaboration improved communication among providers across facilities (including referral hospitals not randomized). Moreover, this regional approach also allowed us to liaise with other organizations working in the area implementing QI or other RMNCH interventions. For optimal impact, we recommend similar efforts to consider this regional network approach covering a referral system in order to maximize outcomes. We recognize, however, that as quality improved in the intervention facilities, more referrals came in from neighboring hospitals and lower level facilities. This increased volume had the potential to compromise the quality of care provided.

Despite these lessons learned, several challenges existed. For example, most of the intervention package was implemented using existing personnel to enable sustainability. Besides the PRONTO mentors and QI consultant, there were no additional staff brought in by the project, which is often part of large intervention studies [33], particularly in LMICs where human resources are limited. This posed an additional challenge in that for Kenyan sites, regular staff transfers occurred every 6 months, from either control to intervention facilities or vice versa, thereby potentially confounding or diluting the effect. The 7-month national nursing strike in Kenya also resulted in substantial workforce loss as well as reallocation of returning nurses to different divisions or facilities. Moreover, need for monetary incentive for completion of the mSCC in Kenya also posed a challenge since it becomes a threat to sustainability of the interventions.

## Conclusion

Publishing more extensive details for complex interventions are important for reproducibility and a more robust understanding of the various elements that contribute to the findings [15, 34]. This is particularly true when adaptation to unique settings and contexts are necessary. Documentation of the study activities based on the TIDieR checklist, or another relevant framework, should be included in the implementation of complex studies to keep track of the key study milestones and lessons learned to enable replication if found to be effective. Such understanding is needed for both replication and the development of stronger interventions in order to tackle the world’s complex health problems.

## Supplementary Information

**Additional file 1: Supplemental Table 1**. The TIDieR (Template for Intervention Description and Replication) Checklist

**Additional file 2: Supplementary Table 2** Equipment and Supplies Provided by Country

## Data Availability

Data sharing is not applicable to this article as no datasets were generated or analyzed during the current study. Materials are available from the study team upon reasonable request.

## Abbreviations

ACS: Antenatal corticosteroids
CRCT: Cluster randomized control trial
DHIS: District Health Information System
DS: Data strengthening
EBP: Evidence-based practices
EmONC: Emergency Obstetric and Neonatal Care
GA: Gestation age
HDREC: Higher Degree Research and Ethical Committee
HIV: Human immunodeficiency virus
IRB: Institutional review board
KEMRI: Kenya Medical Research Institute
KMC: Kangaroo Mother Care
LMIC: Low- and middle-income countries
mSCC: Modified Safe Childbirth Checklist
NGO: Non-government organization
PDSA: Plan-Do-Study-Act
PIP: Program Impact Pathway
PTBi: Preterm Birth Initiative
PTBI-EA: Preterm Birth Initiative East Africa
QI: Quality Improvement
RMNCH: Reproductive Maternal Newborn and Child Health
UCSF: University of California San Francisco
WHO: World Health Organization
