# Supplementary material for: Implementing an intrapartum package of interventions to improve quality of care to reduce the burden of preterm birth in Kenya and Uganda
Source: Implement Sci Commun. 2021 Jan 28;2:10. doi: 10.1186/s43058-021-00109-w (PMC7841990; doi:10.1186/s43058-021-00109-w)
Supplement: Supplementary file 2 — Additional file 2: Supplementary Table 2 Equipment and Supplies Provided by Country [file 43058_2021_109_MOESM2_ESM.docx]

**Supplementary Table 2: Equipment and Supplies Provided by Country**

|  | Kenya | Uganda |
| --- | --- | --- |
| **A. Study-specific supplies provided to ALL FACILITIES** | | |
| - Paper copies of the modified Safe Childbirth Checklist | X | X |
| - PRONTO simulation supplies | X | X |
| - Newborn registers for sick and small newborns |  | X |
| **B. Minimum necessary supplies provided to ALL FACILITIES** | | |
| - Patient charts | X | X |
| - Gestational wheels | X | X |
| - Tape measures | X | X |
| **C. Basic supplies provided to facilities with identified gaps** |  |  |
| - Infant scales | X | X |
| - Preterm-appropriate bag and masks for resuscitation | X | X |
| - Resuscitation tables - Penguin bulb suckers | X  X | X  X |
| **D. Supplemental supplies provided to some facilities based on locally identified need** | | |
| - IV cannula |  | X |
| - Nasogastric tubes |  | X |
| - Common newborn medications |  | X |
| - Gloves | X | X |
| - Phototherapy machines |  | X |
| - Incubator (referral hospital) |  | X |
| - Feeding cups | X | X |
| - Oxygen and fluid infusion sets |  | X |
| - Warming blankets for infant transfer during referral | X |  |
| - Improved warming and lighting systems | X |  |
